# Research breakdowns: A constructive critique of research practice involving grief, trauma and displaced people

**DOI:** 10.1017/gmh.2024.60

**Published:** 2024-05-09

**Authors:** Clare Killikelly, Hannah Comtesse, Franziska Lechner-Meichsner, Johanna Sam, John S Ogrodniczuk

**Affiliations:** 1 Department of Psychiatry, University of British Columbia, Vancouver, BC V6T 1Z3, Canada; 2 Division of Clinical Intervention and Global Mental Health, University of Zurich, Zurich, Switzerland; 3 Clinical and Biological Psychology, Catholic University Eichstaett-Ingolstadt, Eichstaett, Germany; 4 Department of Psychology, Clinical Psychology, Utrecht University, Utrecht, The Netherlands; 5 Department of Educational and Counselling Psychology, and Special Education, University of British Columbia, Vancouver, BC, Canada

**Keywords:** global mental health, epistemic justice, prolonged grief disorder, refugee and displaced peoples, decolonial approach

## Abstract

Impactful research on refugee mental health is urgently needed. To mitigate the growing refugee crisis, researchers and clinicians seek to better understand the relationship between trauma, grief and post-migration factors with the aim of bringing better awareness, more resources and improved support for these communities and individuals living in host countries. As much as this is our intention, the prevailing research methods, that is, online anonymous questionnaires, used to engage refugees in mental health research are increasingly outdated and lack inclusivity and representation. With this perspective piece, we would like to highlight a growing crisis in global mental health research; the predominance of a Global North-centric approach and methodology. We use our recent research challenges and breakdowns as a learning example and possible opportunity to rebuild our research practice in a more ethical and equitable way.

## Impact statement

Here, we explore the challenges of conducting research on refugee mental health and propose a new framework that aims to improve research quality through equitable research practices.

## Introduction

Now more than ever, research on refugee mental health is urgently needed. The refugee crisis is escalating, with 110 million forcibly displaced people around the world, which is up from 79.5 million in 2019 (https://www.unhcr.org/refugee-statistics/, January 2024). The number of people fleeing wars and conflicts in Syria, Afghanistan, the Ukraine and Gaza continues to increase. The suffering and hardship that forcibly displaced people have endured is monumental (Bryant et al., 2023). Researchers and clinicians hope to better understand the relationship between trauma, grief and post-migration factors with the aim of bringing better awareness, more resources and improved support for these communities and individuals living in host countries. As much as this is our intention, the prevailing research methods, that is, online anonymous questionnaires, used to engage refugees in mental health research are increasingly outdated and lack inclusivity and representation. With this perspective piece we would like to highlight a growing crisis in global mental health research; the predominance of a Global North-centric approach and methodology. We use our recent research challenges and breakdowns as a learning example and possible opportunity to rebuild our research practice in a more ethical and equitable way. In this article, we present three sections: (1) the research breakdown of our recent project on refugee mental health research; (2) a review of recent literature and theory proposing a crisis in global mental health research and (3) a new framework for diversity and inclusion in mental health research methods.

### Conceptual clarification: inclusivity and representation in refugee mental health research

Our aim is to share our research journey, ongoing learning and shift toward diverse and inclusive research practices prioritizing the lived experiences of refugees. Here, we outline the specific actions we have taken and plan to take to correct power imbalances, explore our own biases and privileges and disrupt the status quo of grief and trauma research in refugee groups. We have consulted several movements, practices and paradigms to reframe our research methods. Several schools of thought provide a strong foundation for challenging the implicit and explicit assumptions about refugee mental health. Literature on diversity, inclusion, discourse ethics, philosophy of psychiatry, relativist approaches to mental health and the notion of conceptual competence have guided our thinking and choice of framework (Canino et al., 1997; Ryder et al., 2011; Cooke, 2018; Gureje et al., 2019; Khan et al., 2022). The recent literature on decolonizing research practices in the context of refugee and migrant health provides a strong framework for constructively rethinking research methods, specifically in terms of our research questions, recruitment methods and the overall impact of our work (Rivera-Segarra et al., 2022).

Our aim is to align our research process with Rivera-Segarra et al. (2022) decolonial approach to mental health research and to apply this framework to our planned research on grief and trauma involving refugees and displaced people with an important caveat. Although the concept of decolonization fits our aims, particularly in terms of questioning assumptions based in Western-centric thinking, we align with Tuck and Yang’s assertion that true decolonization takes place at the tribal level, outside of the academic sphere (Tuck and Yang, 2012). The focus of this study is the application of a research framework grounded in diversity, inclusion and representation of displaced people.

## Current state of the field: Research breakdowns

In the below example, we highlight a common experience of many research groups working in refugee mental health: difficulties with recruitment and sampling. In 2021, we started a multi-country (Canada and Germany) research project using online questionnaires of mental health outcomes and predictors of grief and trauma in Arabic-speaking refugees (see https://osf.io/n6j32/). Web-based or online questionnaires are a common and well-known research methodology frequently used in the field (Silove et al., 1997; Hassan et al., 2016; Schick et al., 2018). From the start, we experienced difficulties recruiting participants as we relied on typical methods in our field, such as reaching out to cultural brokers i.e. community-based organizations or social media campaigns. In addition, we were confronted with a new, unsettling barrier: the closure of a large social media campaign due to hate comments. Online hate is conceptualized as “the use of aggressive or offensive language, targeting a specific group of people sharing a common property, whether this property is gender, their ethnic group or race, their believes and religion or their political preferences” (p. 5, Castaño-Pulgarín et al., 2021). A systematic review revealed there are different types of online hate, including religious hate speech, racism, political, gendered, terrorism and hate expressions (Castaño-Pulgarín et al., 2021). In our own work, online hate speech included the following social media comments (translated from German): *“The (university) brings its own enemies into the country", “Why don’t they speak German?”, “This is the last thing we need right now”.* These are merely a few examples of the many messages left under our recruitment posters online.

The challenge of recruiting refugee participants to large online studies has been anecdotally noted in recent conferences in the field. Recently, a new guideline paper presented solutions to the problem of refugee recruitment and dropout (10.31234/osf.io/nukv3). It has been difficult to access these vulnerable groups, recruit enough participants and convince stakeholders to take action; now recruitment for our research project was blocked due to online bullying (Hynie et al., 2022).

This research breakdown, that is, cancelation of our online recruitment campaign and subsequent failure to recruit enough participants, elicited serious reflections from our research team. For years, we have written about the urgent need to document, assess and provide support for refugees suffering from mental health disorders (Fazel et al., 2005). Previously, we have asserted that providing data on the prevalence, incidence and course of mental health symptoms in refugees would allow better allocation of resources, better understanding of their specific needs and more culturally appropriate interventions that were effective. Some research groups have successfully moved to more bottom-up methods (Heim and Kohrt, 2019). Our recent research breakdown has prompted our team to reflect on and revise our research methods to move away from online, questionnaire-based prevalence studies. We urgently needed to adapt our methods to meet the growing need and desire for increased inclusivity and representation in our research. Below, we explore a new framework for how to adapt our research methods to meet this need. However, before this framework is presented, the rationale and previous literature examining this need for action are presented.

### Crisis in global mental health research

In the last 3 years, several touchstone commentaries and calls to action have explicitly and attentively outlined a growing crisis in global (mental) health research. In 2020, Lawrence and Hirsch wrote one of many commentaries outlining the persistence of colonial research practices in global health research (Büyüm et al., 2020; Lawrence and Hirsch, 2020; Oti and Ncayiyana, 2021; Khan et al., 2022). They focused on how transnational research partnerships involving randomized controlled trials maintain colonial power imbalances, biases and lack of representation (e.g., composition of study teams, decision-making, authorship). They question the idea of partnership between low- and middle-income countries and high-income countries and if it is ever truly equitable. They suggest three main ways that international research programs can be more equitable: participant experience, expertise, infrastructure and authorship (Lawrence and Hirsch, 2020). These themes are extended in further calls to action and commentaries. For example, Kronick et al. (2021) highlighted three main challenges for global mental health research involving refugee and displaced peoples: *the role of refugees as victims, traditional research practice that recreates powerlessness and lack of translation into practice.* First, in mental health research, refugees primarily play the role of the subject to be studied and are rarely included as collaborators and partners. This perpetuates colonial power imbalances where the refugee participant is seen as unwell, vulnerable, victimized and lacking agency. In line with that, it has been our experience that professionals and volunteers from the majority society who work with refugees often act as gatekeepers. Many are reluctant to share information about research projects with refugees or advise them against participating out of a concern that answering questions about grief or trauma might be too distressing. While every potential study participant has, of course, the right to decide whether or not they want to be involved in a study, refugees are often not given the opportunity to make this choice for themselves and instead are seen as needing protection.

This is a one-sided conceptualization of refugee health, as refugees are also resilient, strong, resourceful and overcoming (Chung et al., 2022; Hirad et al., 2023). ‘Nothing about us, without us’ (Fricker, 2007) advocates for participatory methodologies as a way forward. However, there are real ethical dilemmas that must be considered. There are risks to speaking out and disrupting the status quo; silence is a key strategy for safety and survival for refugee groups (Kronick et al., 2021). From our recent experience, many participants did not answer questions about the duration of their journey and the concrete status of the asylum application, even though we explained our data handling procedures to them in detail. Kronick et al. (2021) suggest careful reflection on the use and purpose of participatory action research. Community-based participatory research (CBPR) places a key emphasis on partnership, research, action and education. Kia-Keating and Juang (2022) explain how CBPR commits to partnering with communities, for example, by clearly outlining the benefits and disadvantages of the co-construction of knowledge, prioritizing the development of relationships with stakeholders and choosing research with social or racial justice impact. ‘Helicoptering’ into communities for our own research benefit (i.e., publications, funding) is no longer tolerable. Kia-Keating and Juang (2022) ask the question about how can research be more open, inclusive and equitable? Shifting the focus from the individual (i.e., researcher, participant) to collaboration and co-construction of knowledge through CBPR is a key step.

Second, traditional research practice (questionnaire, interview based and online data collection) may be recreating experiences of powerlessness and control that displaced people escaped (Kia-Keating and Juang, 2022). What is more, these practices might also resemble the experiences of asylum seekers during their asylum procedure, which is often associated with power imbalance, uncertainty and fear. Mental health research methods and questions often decontextualize the individual and emphasize individual-level variables such as symptoms, physiology, genetics and behaviors instead of systemic, cultural or ecological factors (Kia-Keating and Juang, 2022). Researchers choose the questions, measures and variables, therefore choosing what is prioritized and presented to the world. In academia, the types of research questions (symptoms, markers, quantitative evidence-based) are often driven by funding bodies and structural factors that prioritize certain types of knowledge and research methods. Depending on the cultural group, research questions with a focus on individual symptoms, processes and functioning might be at odds with what participants themselves view as important (Kohrt et al., 2014; Killikelly et al., 2018). This can also lead to a lack of participation and high dropout rates. This reflects another challenge; relocating the research question in a broader socio-ecological framework instead of at the individual level (Kronick et al., 2021). Research questions should include sociological and structural processes. For example, a research question about depression in refugee groups could be expanded to include questions about access to support, help-seeking behavior and impairment in family relationships alongside individual depression symptom measures.

Finally, translating research into practice is a significant challenge for all areas of mental health research. In the case of research involving refugees, key questions emerge about who benefits from this research and what are the long-term plans for support and resourcing typically underfunded organizations and programs (Kronick et al., 2021). Recently, Rivera-Segarra et al. (2022) challenged the predominant use of Western-centric research methods, ‘evidenced-based practice’ and therapeutic models that are largely developed from a system that historically oppresses and exploits less powerful groups. Most global mental health research, including research on refugees in high-income host countries, is led by White, Western-educated clinicians who benefit economically and professionally, while foundational clinical research studies are mostly conducted on White-educated industrialized rich democratic populations (Henrich et al., 2010). Disrupting this current power imbalance in academia means establishing equitable partnerships, but also prioritizing other types of knowledge and mental health practices (epistemic justice). It also means sharing support and resources (pragmatic solidarity), as well as redefining barriers and breaking traditional research norms (sovereign acts) (Rivera-Segarra et al., 2022). Each of these concepts will be discussed in the following sections.

#### The case of grief research and refugee mental health

Prolonged grief disorder (PGD) is a newly recognized mental health disorder that has recently been included in diagnostic manuals used worldwide (Rosner et al., 2021). The majority of research examining grief in refugee mental health groups has focused on establishing prevalence rates, diagnostics and comorbidities of PGD, PTSD and MDD, for example (e.g., Bryant et al., 2020). With the exception of a few studies (Killikelly et al., 2021; Lechner-Meichsner and Comtesse, 2022), very little research has been conducted using a bottom-up, qualitative or participatory action approach.

PGD is the subject of much academic and public debate (Boelen et al., 2020). Critics of PGD have argued that a grief diagnosis is ‘psychiatry’s colonization of grief’ (Bergsmark and Ramsing, 2023). In this view, PGD represents a trend in the Western Euro-centric society of ‘diagnostic culture’ and the psychologization of human experiences (Bergsmark and Ramsing, 2023). Holte Kofod (2017) presents the possibility that the existence of a PGD diagnosis will shape how bereaved people see and experience their grief, that is, they may become more likely to interpret their experience as symptoms of disorder. Others suggest that the establishment of a PGD diagnosis will minimize structural problems and inequalities and dismiss other causes of suffering outside the individual (Bergsmark and Ramsing, 2023). This may be particularly relevant for mental health research involving refugees.

One question that emerges from existing research findings is what high prevalence rates of PGD mean in refugee groups. Rates of PGD are often found to be higher than 50% (Killikelly et al., 2018; Lechner-Meichsner et al., 2024). Currently, PGD can only be diagnosed if the grief response violates the social and cultural norms of the individual’s cultural context. If the majority of people meet the criteria for a disorder of grief, is it really a grief disorder? If grief and trauma are the cultural norm, how useful is a diagnosis? Practically, this means that the PGD diagnosis in these groups has low clinical utility. If every other person meets criteria for PGD, this shifts the usefulness of the PGD diagnosis criteria from detection and assessment at the individual level to large-scale intervention at the societal level. Conceptually, there are questions about the cultural relevance of the PGD diagnostic criteria. High rates of PGD in refugee populations may be detected with our Western-based conceptualization of PGD disorder, but this may not necessarily translate into impairment in functioning, disability or need for intervention. There may be other factors or phenomenological experiences related to grief (e.g., homesickness, loss of culture and ambiguous loss) that may be more relevant and impairing than a PGD diagnosis (Comtesse and Rosner, 2019; Killikelly et al. 2021; Lechner-Meichsner and Comtesse, 2022; Rosner et al., 2022). This points to the need of studying grief in participatory research that enables us to take a viewpoint that is more open than studies with instruments developed in the Global North. For example, a deeper understanding of societal cultural norms surrounding grief and bereavement can provide a starting point for enhancing the potential relevance and clinical utility of the PGD diagnosis in refugee groups and for developing meaningful treatment programs.

## A new framework for inclusion and diversity in refugee mental health research

We come to this work as educated clinicians and researchers from diverse backgrounds and experiences. In Canada, the research team includes nine bachelor-level psychology students of Arabic-speaking backgrounds from diverse regions, including Syria, Jordan, Palestine, Saudi Arabia, Egypt and Lebanon. All are first-generation Canadians (parents migrated to Canada) or migrants. The research team in Germany was composed of two White European female master-level psychology students, one White European male master-level student and support from one colleague (master-level psychologist) from Syria and further support from one student assistant from Syria (master-level social worker).

As we rethink our research agenda, we start by asking: what is our intention in assessing mental health in refugees? What can we practically achieve to this end? Who do our studies benefit? Is there perhaps a more collaborative, acceptable method to assessment?

Based on the approach outlined by Rivera-Segarra et al. (2022) in Table 1, we outline our original research methods and how we plan to modify these methods using a framework based on a decolonial approach. We have redesigned our study to align more closely with three elements from Rivera-Segarra et al. (2022), epistemic justice (inclusivity in all areas related to knowledge, understanding and participation), pragmatic solidarity (resolving power imbalances through material means, equitable sharing of infrastructure and resources) and sovereign acts (self-reflective and team awareness of the current norms and practicing other ways of being in the world).

**Table 1.** Modified research methods following a decolonial approach

Research element	Original design	Redesign	Decolonial element example
Background, rationale	Longitudinal, repeated measures, country comparison of mental health predictors and outcomes	Community–based participatory action research including co–conducting focus groups, key informant interviews, collaboration with community leaders and cultural brokers	Epistemic justice: prioritization of different types of knowledge and sources of research questions
Main objections	Examine the 12–month trajectory of PGD symptoms in refugees. (a) Which factors (e.g., post–migration stressors, lack of social support) maintain PGD symptoms over time? (b) What are the temporal associations between PGD symptoms and PTSD (as well as other symptoms of physical and mental health)?	Establish partnerships with local organizations, key workers through shared goals and values, exploring long–term project planning and shared resources Co–construct research questions, research method and planned outcomes for social change, using a bottom–up approach focused on collaboration and co–creation with refugee participants	Epistemic justice: ensuring that the research questions and objectives emerge from the community
Study design	Three online surveys with *N* = 250 Arabic–speaking refugees in Canada and Germany	Qualitative, semi–structured interviews, focus groups, ongoing self–reflection and team reflection, time for commitment to partnerships	Sovereign acts: designing the project to include time and space for self and team reflection
Sample and recruitment	Inclusion and exclusion criteria • Minimum age = 18 years. • Currently living in Canada/ Germany (in addition: applied for asylum in Germany) • Sufficient knowledge of. A rabic (or German or English) • Sufficient literacy skills in Arabic, and • Willingness to participate in the study and informed consent Online recruitment using social media, listservs, forums, websites	Partnerships with cultural leaders, local organizations, community members and youth Creating safe online spaces for refugee recruitment by working with refugee groups and leaders to develop online content, websites and questionnaires acceptable for this group, for example, turning off comments for online campaigns, using local idioms and language, providing access to local knowledge and resources Consulting refugee youth on online resources, forums or social media outlets for recruitment purposes Shifting from online to locally acceptable and feasible recruitment methods, for example, word of mouth, snowballing method, building on trust and rapport within the community	Pragmatic solidarity: prioritizing the voices and experiences of the community members, inclusivity in recruitment and sharing of space, time and resources across all levels
Planned analysis	Statistical analysis examining prevalence rates, correlations with mental health symptoms, cross country comparison	Framework analysis, local sourced knowledge, cocreating analysis and research questions that benefit the refugee community in the short and long term	Epistemic justice: including all types of knowledge, prioritizing community voices
Outcomes	Mapping of prevalence rates, indicators and predictors of disorder	Immediate impact for the community, for example, empowering local voices, long–term support system, funding, structural changes to mental health support	Pragmatic solidarity: establishing practical structural changes based on community needs and wishes
Funding	Swiss National Science Foundation Post Doc funding CK, proFOR+ fund of the Catholic University Eichstätt–Ingolstadt for HC, Freunde und Förderer der Goethe Universität for FLM	Plan to apply for local Decolonial funding initiatives (UBC), European and Swiss funding sources for qualitative research, aim to sustain collaborations	Pragmatic solidarity: planning for long–term collaborations and sustainable benefits for community members
Authorship	Led by researchers	Research led by community members	Sovereign acts: including non–academics and students in the authorship process

This new framework provides an opportunity to restructure our research methods using a more inclusive and representative approach. Instead of using online questionnaire data on anonymous individual symptoms, we start from the bottom-up by building relationships, rapport and connection with the refugee community. This shifts the focus to the long-term inclusion and collaboration with this community. The shared goals, values and methods used in the research project will be decided from within the community and co-created with researchers and refugees working together. This has the potential to form long-lasting, effective partnerships to make impactful changes in the community. However, this framework is currently a model and is yet to be tested practically. There may be some significant barriers to conducting this type of research given the financial and time constraints of many research projects. Examining the feasibility of this method is the next step of our research project.

## Conclusion

Here, we turn our own research breakdowns into an opportunity to build a better, more inclusive and equitable research program for refugee mental health. By acknowledging common failures in global mental health research, such as the role of refugees as victims, traditional research practice recreates powerlessness and lack of translation into practice. We hope to join a new wave of research that emphasizes epistemic justice, pragmatic solidarity and sovereign acts.

We would like to invite our colleagues in the field to consider the following questions when planning and implementing refugee mental health research: what is our intention in assessing mental health in refugees? What can we practically achieve to this end? Who do our studies benefit? Is there perhaps a more collaborative, acceptable method to assessment? As a preliminary guide and template, Table 1 may serve as a thought exercise for students, researchers and clinicians engaging in mental health research in the global and local sphere.

In conclusion, we would like to emphasize that the suffering of such a great number of people makes it necessary for our research to contribute to changes for the better. This is only truly possible by also changing the status quo of how we conduct this research; by empowering refugee people to conduct their own research to identify mental health needs and supports.
