# Supplementary material for: Research breakdowns: A constructive critique of research practice involving grief, trauma and displaced people
Source: Glob Ment Health (Camb). 2024 May 9;11:e68. doi: 10.1017/gmh.2024.60 (PMC11362994; doi:10.1017/gmh.2024.60)
Supplement: Peer review history

# Editor report: Recommendation: Research breakdowns: A constructive critique of research practice involving grief, trauma and displaced people — R0/PR2

**DOI:** 10.1017/gmh.2024.60.pr2

**Affiliations:** Ponce Health Sciences University, Puerto Rico

No accompanying comment.

# Editor report: Decision: Research breakdowns: A constructive critique of research practice involving grief, trauma and displaced people — R0/PR3

**DOI:** 10.1017/gmh.2024.60.pr3

**Affiliations:** Johns Hopkins University Bloomberg School of Public Health, United States

No accompanying comment.

# Editor report: Recommendation: Research breakdowns: A constructive critique of research practice involving grief, trauma and displaced people — R1/PR5

**DOI:** 10.1017/gmh.2024.60.pr5

**Affiliations:** Ponce Health Sciences University, Puerto Rico

Please, address the following minor suggestions: 

1. In the intro, the authors mention Gaza in a long list of countries from which people are fleeing-- technically people are not leaving the country, they are IDPs not refugees.

2. The phrase “research breakdown” was a little confusing in the first read, but then it made sense later. Consider revising if others had a similar reaction.

3. The transition to the section on grief is still a bit jarring, consider explaining that it will be used to illustrate the points related to decolonial approach.

4. A final edit for grammar and sentence structure is recommended for the final draft. Its so difficult when there are all the track changes!

# Editor report: Decision: Research breakdowns: A constructive critique of research practice involving grief, trauma and displaced people — R1/PR6

**DOI:** 10.1017/gmh.2024.60.pr6

**Affiliations:** Johns Hopkins University Bloomberg School of Public Health, United States

No accompanying comment.
